# Coral community response to bleaching on a highly disturbed reef

**DOI:** 10.1038/srep20717

**Published:** 2016-02-15

**Authors:** J. R. Guest, J. Low, K. Tun, B. Wilson, C. Ng, D. Raingeard, K. E. Ulstrup, J. T. I. Tanzil, P. A. Todd, T. C. Toh, D. McDougald, L. M. Chou, P. D. Steinberg

**Affiliations:** 1 SECORE International, 40 Jalan Anjung 5, Horizon Hills, Nusajaya 79100, Johor, Malaysia; 2 Centre for Marine Bio-Innovation, School of Biological, Earth and Environmental Sciences, University of New South Wales, Sydney, NSW 2052, Australia; 3 Department of Biological Sciences, National University of Singapore, Singapore 117543; 4 National Biodiversity Centre, National Parks Board, Singapore Botanic Gardens, Singapore 259569; 5 Department of Biology, University of Bergen, Thormøhlensgate 53B, 5020 Bergen, Norway; 6 National University of Singapore Environmental Institute, National University of Singapore, Singapore; 7 Cooper Marine, PO Box 60, Mosman Park WA 6912, Australia; 8 Earth Observatory of Singapore, Nanyang Technological University, Singapore 639798; 9 Tropical Marine Science Institute, National University of Singapore, Singapore 119227; 10 iThree Institute, University of Technology Sydney, Australia; 11 Singapore Centre on Environmental Life Sciences Engineering, Nanyang Technological University, Singapore; 12 Sydney Institute of Marine Science, Mosman, NSW 2088, Australia

## Abstract

While many studies of coral bleaching report on broad, regional scale responses, fewer examine variation in susceptibility among coral taxa and changes in community structure, before, during and after bleaching on individual reefs. Here we report in detail on the response to bleaching by a coral community on a highly disturbed reef site south of mainland Singapore before, during and after a major thermal anomaly in 2010. To estimate the capacity for resistance to thermal stress, we report on: a) overall bleaching severity during and after the event, b) differences in bleaching susceptibility among taxa during the event, and c) changes in coral community structure one year before and after bleaching. Approximately two thirds of colonies bleached, however, post-bleaching recovery was quite rapid and, importantly, coral taxa that are usually highly susceptible were relatively unaffected. Although total coral cover declined, there was no significant change in coral taxonomic community structure before and after bleaching. Several factors may have contributed to the overall high resistance of corals at this site including *Symbiodinium* affiliation, turbidity and heterotrophy. Our results suggest that, despite experiencing chronic anthropogenic disturbances, turbid shallow reef communities may be remarkably resilient to acute thermal stress.

Coral bleaching refers to the paling or whitening of shallow water reef corals that results from the loss of symbiotic dinoflagellate algae (known as zooxanthellae) or their algal pigments^1^. Bleaching is a stress response, leading to sub-lethal damage and/or partial or whole colony mortality for the coral host, and can be induced by several factors^1^. Large-scale bleaching episodes, however, are strongly correlated with elevated sea temperatures and may be exacerbated by high irradiance^2^. Major bleaching events driven by thermal anomalies have caused widespread and catastrophic mortality of corals and are considered one of the main drivers of global reef degradation^3^. Bleaching severity, at the level of the coral assemblage, varies substantially as a consequence of the severity of thermal stress^4^, the thermal history of the site e.g.^5^, local environmental factors that affect irradiance (such as cloud cover and turbidity)^6^,^7^,the type and diversity of symbiotic algae present in the coral host tissue^8^,^9^ and the taxonomic community structure of the coral assemblage^10^.

Of these factors, the role of the symbiont is probably one of the most intensively researched areas in climate change studies of corals, because certain zooxanthellae types are associated with greater thermal tolerance^9^. The taxonomic composition of the coral assemblage is another strong driver of variation in bleaching at a reef scale^10^. This is because consistent differences have been documented in the susceptibility of coral taxa to bleaching^10^,^11^,^12^; but see Guest *et al.* (2012)^13^. For example, in most studies to date, branching colonies of the genera *Acropora* and *Pocillopora* have been reported to bleach much more severely relative to other growth forms and coral genera, particularly slow growing massive species^10^,^11^,^12^. This observation has led to the widespread classification of fast growing branching species as “losers” in the face of global warming^11^, at least in the short term^14^. The classification of corals as winners or losers does not, however, take into consideration the capacity for normally susceptible species to adapt and/or acclimatise to thermal stress^15^,^16^,^17^. Not only do different coral taxa vary in their bleaching responses, but they also differ in their ability to recover from bleaching. For example, in a study of individually tagged colonies of four species on the Great Barrier Reef (GBR) following bleaching, 88% of *Acropora hyacinthus* and 32% of *A. millepora* colonies (both fast growing branching species) died, compared to whole colony mortality in only 13% of *Platygyra daedalea* and 0% of *Porites lobata* (both slow growing massive species)^18^. Most surviving *P. daedalea* and *P. lobata* colonies lost some tissue through partial colony mortality but this was rare in the *Acropora*; colonies either survived intact or died completely^18^. The physiological mechanisms that lead to greater susceptibility among certain coral taxa and growth forms are not fully understood^19^, but may be associated with differences in the type of symbiont hosted^9^, metabolic rates^20^, tissue thickness^11^, mass transfer rates^21^ and heterotrophic feeding capacity^22^.

Differential bleaching severity among taxa has important ecological implications because it influences the potential of species to adapt to future thermal stress by natural selection^17^. Susceptible taxa, such as *Acropora* and *Pocillopora*, have relatively fast growth rates^23^ and become sexually mature within two to three years^24^,^25^, life history traits which predispose these genera to adapt more rapidly^17^. Thus, one might expect a more rapid adaptive response on reefs dominated by fast growing susceptible species (providing that at least some of the coral population recovers following bleaching)^16^. The incidence of partial versus full colony mortality within a coral community following bleaching also has implications for the rate at which a reef recovers following a disturbance. Reefs dominated by species with “generalist” life history traits (i.e., stress tolerance *and* rapid growth rates)^26^ may recover coral cover rapidly via regrowth of remnant colonies, rather than relying on recruitment of new colonies from larvae^27^,^28^.

Understanding the potential of reef assemblages to recover from disturbances (such as bleaching events) is becoming increasingly important as the frequency and magnitude of anthropogenic impacts affecting reefs increase^29^. Temperature-induced mass coral bleaching events are predicted to become more frequent^30^ and reef coral assemblages will undoubtedly change in terms of their taxonomic composition over the next few decades^29^. While many studies report on broad, regional scale responses to thermal stress during bleaching events (e.g., proportion of corals bleached)^31^, fewer examine variation in susceptibility among taxa and change in coral community structure before, during and after bleaching on individual reefs^11^,^14^,^15^,^32^,^33^,^34^. Such detailed studies are needed if we are to understand the effect that repeated thermal anomalies will have on community structure and reef resilience. Furthermore, less is known about the capacity for resistance and recovery on reefs that experience severe chronic disturbances such as elevated sedimentation and turbidity^32^. It is often assumed, however, that these reefs will be less resilient to additional disturbances compared to reefs that are managed effectively or reefs far from anthropogenic influences^27^,^35^.

In 2010, corals throughout the Indian Ocean and Southeast Asia experienced higher than normal water temperatures leading to extensive coral bleaching at many reef sites (http://coralreefwatch.noaa.gov/satellite/bleachingoutlook/index.html). Severe bleaching was reported in Singapore and Malaysia^13^,^36^, Thailand^37^,^38^,^39^, Indonesia^40^,^41^,^42^, Brunei, Cambodia, Philippines and Vietnam^43^. In an earlier publication, Guest *et al.* (2012)^13^, reported an unusual reversal in bleaching susceptibility among coral taxa and contrasting bleaching responses among sites in Singapore, Malaysia and Sumatra during 2010. To further examine the capacity for resistance of Singaporean reefs, here we report on the bleaching responses of corals at a reef site south of mainland Singapore during (June, July) and immediately after (October) the 2010 bleaching event. The aims of this study were to: a) estimate the capacity for resistance to thermal stress by quantifying overall bleaching severity during and after the event, b) compare relative bleaching susceptibility among taxa during and after the event, and c) examine the effect of the bleaching event on coral cover and coral taxonomic community structure. We also carry out preliminary analysis of *Symbiodinium* clade affiliation for two common coral species to assess the possible role of different symbiont clades in thermal tolerance.

## Results

**Figure 1:**
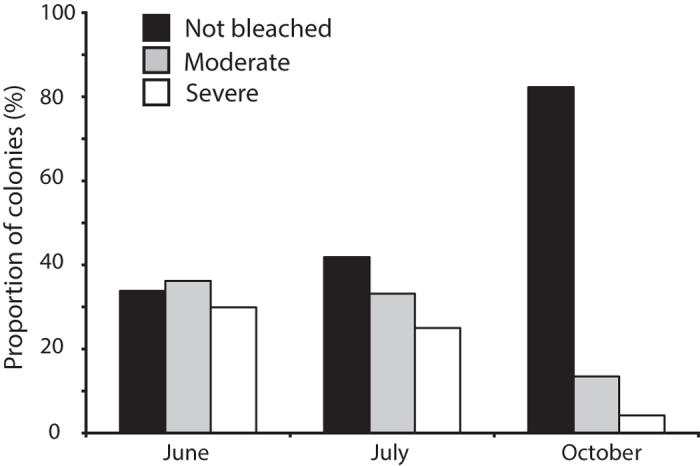
Overall coral bleaching severity in June, July and October in terms of proportion (%) of colonies not bleached, moderately bleached and severely bleached (corresponding to categories 1, 2 and 3 respectively, see methods).

A total of 597, 542 and 491 colonies were surveyed in June, July and October 2010 respectively at a fringing reef on the western side of Pulau Satumu (Raffles Lighthouse, 1°09′35.09″N, 103°44ʹ24.09″E). Surveys of taxa susceptibility of all genera revealed that an estimated 66% in June and 58% in July of colonies were bleached (Fig. 1). In June an estimated 36% of colonies were moderately bleached and 30% were severely bleached, whereas in July there was already some evidence of recovery with 33% moderately and 25% severely bleached (Fig. 1). In contrast, in October only 18% of colonies surveyed were bleached, with only 4% severely bleached and 14% moderately bleached (Fig. 1). A total of 30 taxa were surveyed during all three sampling occasions in 2010. Bleaching severity varied greatly among populations within each sampling occasion and within populations between sampling occasions. The proportion of colonies bleached varied from 0–100% among genera in all survey months, however, for most taxa, bleaching severity declined markedly between June and October (Table 1). For example, in June and July, 22 of 30 taxa surveyed had ≥50% of colonies bleached, whereas in October only 4 taxa had ≥50% of colonies bleached (Table 1).

**Figure 2:**
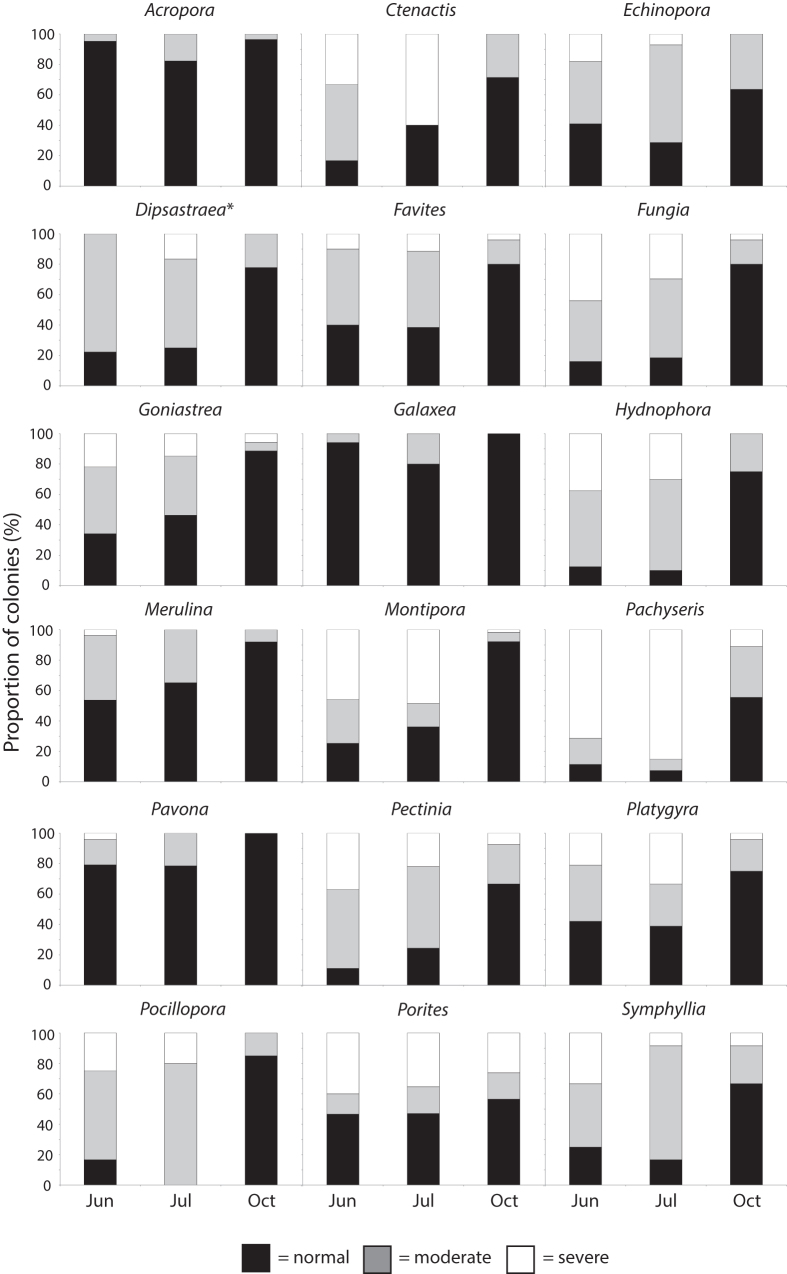
Proportion (%) of colonies not bleached (black bars), moderately bleached (gray bars) and severely bleached (white bars) for all genera that had at least 5 colonies surveyed on all three survey occasions. *This genus was formerly *Favia*.

**Figure 3:**
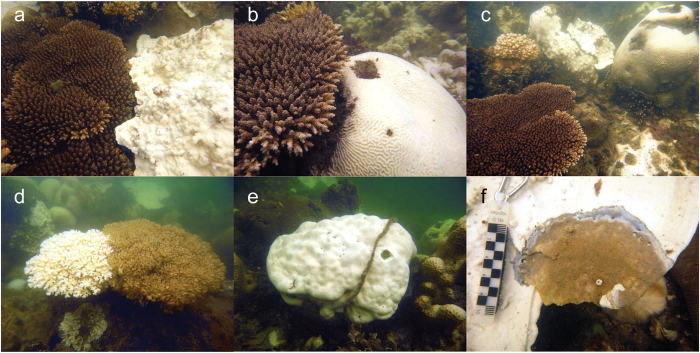
Bleaching at Pulau Satumu in July 2010. (**a**) healthy colony of *Acropora tenuis* next to severely bleached colony of *Montipora*, (**b**) healthy colony of *A. tenuis* next to severely bleached colony of *Platygyra* sp., (**c**) healthy colony of *A. hyacinthus* with severely and partially bleached massive and encrusting corals, (**d**) severely (left) and partially (right) bleached colonies of *Pocillopora damicornis*, (**e**) severly bleached colony of *Porites* (massive) and (**f**) partial mortality and disease in bleached colony of *Montipora* sp. Photos: JR Guest.

We estimated a bleaching index (BI)^12^ for each taxa to compare bleaching responses among taxa (see methods). Considering only taxa for which at least 5 colonies were sampled on all 3 occasions, 4 coral genera had consistently low susceptibility to bleaching (i.e., BI < 30) throughout the study period (Supplementary Table S1 online, Fig. 2). These included *Acropora*, *Galaxea*, *Pavona* and *Merulina* (Supplementary Table S1 online, Fig. 2). The genus *Acropora* was the least affected among all genera, with 82% of colonies surveyed in July (at least 13 species surveyed) unaffected by bleaching with the remainder either pale or partially bleached and none severely bleached (Table 2, Figs 2 and 3a–c). A further 7 genera had relatively moderate susceptibility to bleaching (i.e., BI from 30–59) in both June and July, but of these, all but one (*Porites*) had low severity (BI < 30) by October (Supplementary Table S1 online, Fig. 2). All colonies of *Porites* surveyed in June and July were massive (primarily *P. lutea* and *P. lobata*) or submassive (*P. rus*). Only one colony of branching *Porites* (possibly *P. cylindrica*) was surveyed in October and it had suffered severe partial mortality (80% of colony), suggesting it had experienced bleaching-related mortality. A further 8 genera had relatively high bleaching susceptibility (i.e., BI ≥ 60) in June and/or July, but again, all had low severity (i.e., BI < 30) by October (Supplementary Table S1 online, Fig. 2). For example, all *Pocillopora* colonies (predominantly *P. damicornis*) were bleached, with 80% moderately and 20% severely bleached in July, but by October only 15% remained moderately bleached (Supplementary Table S1 online, Figs 2 and 3d). The massive taxa *Porites*, on the other hand recovered less quickly with 43% of colonies moderately or severely bleached in October, compared to 53% in July (Supplementary Table S1 online, Figs 2 and 3e).

In October 2010, 15 out of 25 genera surveyed had colonies exhibiting partial mortality. The most severely affected genera (i.e., genera with >50% of colonies exhibiting partial mortality) were *Oulophyllia*, *Lobophyllia*, *Goniastrea* and *Merulina* with 100, 80, 67 and 60% of colonies respectively having some partial mortality (Table 2). The extent of partial mortality was also high for some colonies of the genus *Dipsastraea* (formerly *Favia*), with 25% of colonies having between 51 and 75% partial colony mortality (Table 2). No colonies of either *Acropora* or *Pocillopora* were recorded to have partial colony mortality, whereas 23% of *Porites* colonies surveyed had low levels (i.e., ≤25%) of partial colony mortality.

Average coral cover declined from 72.2 ± 11.1% (mean ± SD) in 2009 to 50.7 ± 8.4% in 2010, but had partly recovered to 56.0 ± 9.1% by 2012. A total of 36 coral genera were surveyed before, during and after the bleaching event in 2009, 2010 and 2012 (Supplementary Table S2 online). While there were changes in relative cover of certain taxa before and after bleaching, there was no significant shift in coral taxonomic community structure among years (One-way ANOSIM, Global R −0.08) suggesting that most differences in relative cover of taxa among years were a result of natural temporal variation. It is worth noting, however, that of the 8 taxa that had high susceptibility to bleaching in June/July, five showed decreases in relative abundance after bleaching (*Pachyseris*, *Fungia*, *Pectinia*, *Hydnophora* and *Montipora*) (Supplementary Table S1 online). In contrast, only one of the four taxa (*Pavona*) that showed low bleaching susceptibility had declined in relative abundance between 2009 and 2012 (Supplementary Table S2 online). The taxa that had the highest bleaching index in October (*Porites*) also had the greatest decrease in relative abundance between 2009 and 2012 (Supplementary Table S2 online). Relative cover of *Acropora* remained unchanged before and after bleaching, while relative cover of *Pocillopora* increased nine-fold (Supplementary Table S2 online).

An investigation of coral symbiont diversity using internal transcribed spacer region 2 (ITS2) tag pyrosequencing (Supplementary Table S3 online) from two coral species revealed that whilst sequences related to *Symbiodinium* clades C and D were both found, clade D predominated in the tissues of both *Pocillopora damicornis* and *Platygyra sinensis*. In five of the six corals sampled, clade D was the only symbiont type present, whilst clade C was only found in one sample of *P. sinensis*. Of the 20 distinct operational taxonomic units (OTU) identified, ten were assigned to subclade D1 and two to subclade C3 (both found in one colony of *P. sinensis*) based on the top BLAST+ hits^44^ to the GeoSymbio database^45^. It was not possible to assign the remaining seven OTUs to subclade level but they all belonged to clade D.

## Discussion

Between May and August 2010 Singapore’s reefs underwent a major bleaching event (i.e., >50% of colonies bleached) as a result of a thermal anomaly^43^. Despite severe bleaching at one Singaporean site (Pulau Satumu) in 2010, post-bleaching recovery appeared to be rapid and coral taxa that are usually highly susceptible (e.g., *Acropora* and *Pocillopora*) were relatively unaffected^13^. Surveys of four other sites around Singapore’s Southern Islands found similar reef-scale bleaching responses, suggesting that Pulau Satumu was representative of the broader coral community^43^. Surveys to estimate bleaching-associated coral mortality carried out in October 2010 at Pulau Satumu revealed that an estimated 4% of colonies had died recently^13^. The majority of the change seen in the proportion of bleached and healthy colonies between June and October was, therefore, due to colonies recovering from bleaching and not as a result of colonies bleaching and subsequently dying. Bleaching-associated mortality on other Singaporean reefs (reported to be <10% of colonies) was much lower than reported at other locations in Southeast Asia, particularly at sites in the Andaman Sea and Gulf of Thailand where close to half of the colonies surveyed died following bleaching^38^,^39^,^42^,^43^, but similar to reports from sites in Peninsular Malaysia (Tioman Island), Indonesia (e.g., Wakatobi, Bali), Brunei, Sabah and Sarawak^43^.

In addition to relatively high overall resistance of corals at Pulau Satumu to bleaching, the taxonomic hierarchy of susceptibility among coral genera was unprecedented. In all studies prior to 2010 there has been a consistent pattern of susceptibility among coral taxa, with branching *Acropora* and *Pocillopora* being more susceptible to thermal stress than massive growth forms^10^,^11^,^12^,^18^. In contrast, in the present study, *Acropora* were the least affected of all taxa, with 5, 18 and 4% of colonies moderately bleached in June, July and October, respectively. In contrast, most surveyed colonies of *P. damicornis* were bleached in June and July (83 and 100% respectively), however, only 15% of colonies remained moderately bleached in October. No recently dead colonies of *Pocillopora* and only one recently dead colony of *Acropora* were recorded during surveys in October 2010 (data from Guest *et al.* 2012)^13^ therefore we can conclude that these, normally susceptible, genera were largely resistant to bleaching-associated mortality at this reef during the 2010 thermal anomaly. In contrast, massive species and other growth forms and taxa behaved normally, i.e., they tended to bleach moderately and recover relatively slowly^18^. For example a high proportion of surveyed *Porites* colonies (43%) were still moderately or severely bleached in October.

Surveys of coral benthic structure showed decreases in overall coral cover after the bleaching event; however, taxonomic community structure did not change significantly. There were no decreases in relative abundance of *Acropora*, but there were increases in relative abundance of *Pocillopora* and decreases in relative abundance of *Porites*. For *Porites*, this was not as a result of species with branching morphologies bleaching and dying, as the majority of *Porites* surveyed at Pulau Satumu were either massive or submassive morphologies (e.g., *P. lutea*, *P. rus*). A similar pattern, i.e., relatively low susceptibility to bleaching of *Acropora* corals, was also reported for sites in Indonesia (e.g., Wakatobi)^40^ and Malaysia (e.g., Tioman Island)^13^, indicating this unusual taxonomic reversal in susceptibility was not restricted to the reef at Pulau Satumu. To the best of our knowledge, the event in 2010 is the first time such a response has been reported for these usually highly susceptible taxa during a major thermal bleaching event. In light of these results, there is a clear need to re-evaluate the current paradigm regarding winners and losers among corals in the face of climate change.

Several possible explanations exist for the overall high resistance to bleaching at Pulau Satumu in 2010. Firstly, Singapore’s corals may host relatively thermally tolerant symbiont types. Currently published accounts of symbiont association from Singaporean reefs only exist for five species of zoantharian which host C1/C3, C15/C91 and clade D derived *Symbiodinium*^46^. For hard corals in Singapore there are currently no published accounts of symbiont diversity for a wide range of species. However, limited sampling from two species that were relatively tolerant to thermal stress in 2010 (*P. damicornis* and *P. sinensis*) using ITS2 tag pyrosequencing, reveal that whilst sequences related to *Symbiodinium* clades C and D were present, clade D predominated (see Supplementary Table S3 online for details). Taxonomic assignment to subclade level was possible for just over half of the OTUs found and these most closely matched (≥98% sequence identity) subclades C3 or D1. The inability to classify the remaining OTUs to subcladal level based on current *Symbiodinium* ITS2 sequence databases suggests that they may represent novel subclades within clade D. Whilst the diversity of coral symbiont clades has historically involved the analysis of interspecific variations in ITS2 data, the recent discovery of extensive intra-specific sequence variation among ITS regions in free-living dinoflagellates^47^ means that further studies involving additional marker genes, such as *psbA* mini circles^48^,^49^ might offer improved subcladal resolution, in lieu of full chloroplast genome sequences^50^. Hosting some *Symbiodinium* clade D types can confer a limited thermal tolerance on coral hosts^9^,^51^, but prevalence of *Symbiodinium* type D is also closely associated with reduced water clarity (as is the case for Singapore)^52^. Previous work suggested that C3 type zooxanthellae were relatively thermally sensitive^53^, although more recent work found this *Symbiodinium* type to be prevalent even in regions with extreme temperature variations^54^. The current, limited availability of samples from Singapore make it difficult to conclude definitively that symbiont clade affiliation is responsible for the high thermal tolerance of Singapore’s corals, however a detailed study of clade types from a wider range of coral species is currently underway.

Secondly, high turbidity, which currently characterises Singapore’s coastal waters may provide a degree of protection from light stress during thermal anomalies. In combination with high temperatures, elevated levels of irradiance can lead to coral bleaching^2^,^7^ and when irradiance is lowered during thermal anomalies (e.g., by cloud cover) corals bleach less severely^6^. Thirdly, it is conceivable that high concentrations of suspended particulate matter (SPM) present in Singapore’s coastal waters, e.g., ranging from 9–16 mg l^−1 ^55, may have provided an opportunity for corals to obtain a higher proportion of their energy requirements from heterotrophy during periods of thermal stress^22^.

A final possible explanation for the level of bleaching resistance at Pulau Satumu is that corals at this site possess greater tolerance to acute temperature fluctuations due to prior acclimatisation, i.e., phenotypic changes by an individual organism in response to fluctuations in natural conditions^56^. It is well established that bleaching is induced when temperature increases significantly above the long-term mean annual maximum^57^ (and references within) and that corals living at higher average ambient temperatures have higher thermal tolerances than those living at lower temperatures^58^. On reefs with similar mean temperatures but differences in overall temperature fluctuations (i.e., differences in standard deviation around the mean), corals that experience the larger fluctuations may also have greater thermal tolerance^56^. Support is provided for this explanation by the fact that annual SST variability (i.e., standard deviation of the mean) is ~40–52% higher at sites in the South China Sea where overall bleaching severity was lower in 2010 compared to sites in the Andaman Sea (this is despite similar levels of thermal stress in 2010 and similar long term average SSTs)^13^.

While the above may explain the overall resistance of corals at Pulau Satumu to bleaching, they do not explain the unprecedented hierarchy of taxonomic susceptibility (e.g. low susceptibility for *Acropora* and *Pocillopora*) seen at this and other sites in Southeast Asia^13^,^40^. A parsimonious explanation for the reversal in the normal hierarchy of susceptibility at certain sites in 2010 is that removal of susceptible individuals from populations that bleached during previous episodes (e.g., 1997–1998)^31^, followed by reproduction and successful recruitment of the remaining, more thermally tolerant individuals, has led to adaptation through natural selection^13^. High variability within populations in response to thermal stress provides a very strong selective pressure. Due to the life history traits of *Acropora* and *Pocillopora*–i.e., fast growth rates^23^, onset of sexual maturity within 2–3 years^24^,^25^ and high rates of whole colony mortality following thermal stress^18^–these taxa are the most likely to adapt in a rapidly changing environment^17^. This hypothesis is supported by the observation that these genera were generally less susceptible to bleaching at certain sites in Southeast Asia that bleached during the last major episode in 1998, whereas they suffered high levels of bleaching-associated mortality in 2010 at sites that did not bleach in 1998^13^. Furthermore, other studies carried out on reefs over successive bleaching events that have documented increasing thermal tolerance and declining rates of bleaching-induced mortality in usually susceptible taxa over successive bleaching events^15^,^34^,^59^. Although, mass bleaching was reported for Singapore in June and July 1998^31^, nothing is known about differences in taxa susceptibility for the reef at Pulau Satumu during that event. At Indonesian sites close to Singapore (e.g., Riau Islands ~200 km south of Singapore)^60^, *Acropora* was reported to be “*particularly susceptible to bleaching when compared to massive coral species*” in 1998^60^. Another major bleaching event was documented at sites in the Java Sea in 1983 61,62 that resulted in catastrophic mortality of branching corals including *Acropora*. Intriguingly, Suharsono^60^ also noted an increase in tolerance of reef flat *Acropora* corals at one site (Pari Island) between bleaching events in 1983 and 1998 and suggested this was evidence of an adaptive response.

The response of corals at Pulau Satumu to thermal stress has important implications for resilience. Surveys of benthic community structure carried out in 2009 and 2010 showed a reduction in overall cover of corals from ~73–51% at this site, however, there was no significant change in coral taxonomic community structure before and after the bleaching event in 2010. In the present study, decreases in relative cover (%) at Pulau Satumu were greatest for the coral taxa *Porites*, *Pavona*, *Pachyseris*, *Montipora* and *Echinopora* between 2009 and 2012. With the exception of *Porites*, which predominantly has massive growth forms in Singapore, these taxa have foliose or encrusting growth forms. Considering that there was little evidence of whole colony mortality following the 2010 bleaching (i.e., ~4% of colonies surveyed)^13^, but 15 coral genera experienced partial colony mortality, it is likely that the observed loss of coral cover in 2010 was primarily due to colonies shrinking in size. Indeed, during surveys in October we regularly noted partial mortality due to disease on bleached colonies (e.g., *Montipora*, Fig. 3f). Singapore does not experience typhoons and major coral predators (e.g., *Acanthaster planci*) have not been documented locally, thus the 2010 bleaching event was the only major natural disturbance affecting Singaporean reefs during the study period. We cannot, however, rule out the possibility that other anthropogenic disturbances (e.g., boat groundings and anchor damage from recreational vessels) also contributed to changes in coral cover between 2009 and 2012 at Pulau Satumu. The processes of regrowth of remnant colonies and recruitment of larvae both contribute to the time taken for coral cover to recover to pre-disturbance levels on reefs, but recovery rates vary among habitats and are highly dependent on the composition of surviving coral communities^27^. Measured linear extension rates for foliose and massive species (e.g., *Merulina ampliata* ~25 mm yr^−1^, *Porites lutea* ~18 mm yr^−1^)^63^,^64^ at Pulau Satumu are within the normal range for scleractinians^23^,^65^. In contrast, estimated rates of settlement of new coral spat at this site are low relative to other Indo-Pacific reefs^66^,^67^. While recovery of coral cover from recruitment of new individuals via sexual reproduction can be a relatively slow process on reefs (i.e., decades), recovery via regrowth of remnants can happen within a period of a few years^27^,^28^, suggesting potential for rapid recovery at this site. Indeed, the reef at Palau Satumu appeared to be on a rapid recovery trajectory, with coral cover increasing from ~50% in 2010 to ~56% in 2012.

In this study we show a remarkable level of resistance to, and recovery from, a major thermal coral bleaching event at a chronically impacted Indo-Pacific reef site. Furthermore, we document a reversed pattern of bleaching susceptibility where usually highly susceptible taxa were relatively unaffected by bleaching. These results suggest an underappreciated resilience in disturbed impacted reef systems and that corals that have been classified as losers in the face of climate change may have a greater capacity for adaptation and/or acclimatization than previously supposed.

## Methods

### Study site

Pulau Satumu is situated approx. 13 km from mainland Singapore and is the southernmost reef within Singapore’s territorial waters. The western fringing reef at Pulau Satumu is approx. 250 m long and 20–30 m wide (i.e., from shore to the base of the reef slope). Singapore’s marine environment is characterised by high levels of sedimentation, turbidity and eutrophication as a result of extensive historical coastal development and ongoing dredging of shipping channels^68^. Although heavily impacted, most of Singapore’s islands have fringing reefs with relatively diverse coral communities, home to over 250 documented scleractinian species^69^. Due to its distance from the mainland, Pulau Satumu has the lowest rates of sedimentation and suspended solids of studied Singaporean reefs^70^. This site also has the highest hard coral cover (>50% total cover) and greatest coral species richness among Singaporean reefs^69^,^70^. While it is one of the least impacted sites in Singapore, levels of sedimentation (~15 mg cm^2^ d^−1^) and suspended solids (~10 mg l^−1^) still exceed values considered normal for coral reefs^55^. The coral community at Pulau Satumu is dominated by coral genera with foliose, encrusting, massive and sub-massive growth forms including *Montipora*, *Merulina*, *Platygyra*, *Echinopora* and *Pachyseris*^66^. This site also has one of the only accessible reefs within Singapore’s territorial waters possessing an assemblage of *Acropora* and *Pocillopora* (primarily *P. damicornis*) corals suitable for meaningful ecological studies^69^.

### Surveys of bleaching susceptibility

Extensive bleaching of corals and other zooxanthellate taxa (e.g., anemones, zoantharians) at multiple sites south of Singapore was recorded in May 2010 43. Coral bleaching is triggered when sea surface temperatures (SST) exceed a climatological maximum monthly mean (MMM) for extended periods. The extent of thermal stress is typically expressed in terms of degree heating weeks (DHW)^71^. In Singapore, remotely sensed data derived from the Pathfinder dataset of the US National Oceanic and Atmospheric Administration indicated that SSTs rose above the climatological MMM of 29.86 °C in mid-April 2010 and remained elevated until at least mid-August 2010^13^. During this period, maximum DHW of 12.02 °C weeks (i.e., accumulation of thermal anomalies at temperatures >29.86 °C) occurred in mid-July^13^, although this was based on a slightly modified version of the most commonly used DHW method (i.e., when thermal anomalies begin to accumulate at ≥1 °C above MMM). The level of thermal stress reported for Singapore was similar to that of other sites in Southeast Asia, including Malaysia and Sumatra^13^. For the present study, surveys of bleaching susceptibility were carried out on June 15, July 5 and October 4 2010, i.e., +8, +12 and + 25 weeks after sea temperatures exceeded MMM in Singapore.

On survey occasions in June and July, coral colonies within four haphazardly placed 30 m × 1 m belt-transects were surveyed at depths between ~2 and 5 m along the reef flat and upper reef slope on the western side of Pulau Satumu. Survey data in October were collected using methods described in Guest *et al.* (2012)^13^. Ten replicate 2-metre radius survey plots were selected haphazardly and all colonies within each plot were surveyed. Although the survey methods in June/July and October differed, the total area surveyed was similar (120 m^2^ in June/July and 126 m^2^ in October). In addition to the transects and survey plots, in July and October a wider survey of the genus *Acropora* was carried out during a 40-minute random swim, in which all *Acropora* colonies encountered were surveyed. All colonies within each transect or survey plot were identified to genus level and bleaching status was recorded as follows: 1) healthy = no bleaching; 2) moderately bleached = colony pale or less than 50% of surface area bleached and; 3) severely bleached = colony greater than 50% bleached^10^. A bleaching index (BI)^12^ was estimated as follows:


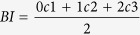


where c1 to c3 are the three coral status categories expressed as the proportion of colonies (%) surveyed arranged in order from not bleached to severely bleached. This differs from the bleaching and mortality index (BMI)^12^ as it does not include recently dead colonies.

### Coral cover, community structure and partial colony mortality

Coral cover and taxonomic community structure were characterized by SCUBA divers using the line intercept method in September 2009, 2010 and 2012 (i.e., one year before bleaching, during bleaching and two years after bleaching). During each survey, five replicate 20 m transects were laid parallel to the reef crest at a depth of ~3–4 m and each transect was separated by a distance of approximately 5 m. The genera and growth forms of all hard coral colonies encountered along each transect were recorded. Non-metric multidimensional scaling (nMDS) ordinations and one-way ANOSIM based on Bray-Curtis similarities of square-root transformed data were produced in PRIMER v6 to examine changes in coral community structure among years. Surveys to estimate the proportion of colonies with recent partial colony mortality were carried out in October 2010 along the same 5 transects described above. Any coral colonies beneath the transect line were counted and identified to genus. Partial colony mortality was also noted, if present, and the proportion (%) of the colony surface area that was dead was estimated by eye and recorded in one of six categories as follows: 1 = no partial mortality, 2 = 1–10% of colony, 3 = 11–25%, 4 = 26–50%, 5 = 51–75%, and 6 = 76–100% of colony.

### Analysis of Symbiodinium types in two coral species

Opportunistic sampling of corals carried out in 2012 from reef sites in Singapore allowed us to assess the dominant clades of *Symbiodinium* found in two species of coral: *Platygyra sinensis* and *Pocillopora damicornis*. Three independent tissue samples of *P. sinensis* and one sample of *P. damicornis* were collected from Pulau Satumu (the study site used for the bleaching surveys), while the other two samples of *P. damicornis* were collected from Kusu Island (a reef site approx. 15 km east of P. Satumu) in December 2011 (18 months after the first bleaching survey) (For a full description of methods see Supplementary Material).

## Additional Information

**How to cite this article**: Guest, J. R. *et al.* Coral community response to bleaching on a highly disturbed reef. *Sci. Rep.* **6**, 20717; doi: 10.1038/srep20717 (2016).

## Supplementary Material

Supplementary Information

## Figures and Tables

**Table 1:** Proportion of colonies bleached in all taxa that were surveyed in June, July and October.

Genus	June		July		October	
	Bleached (%)	n	Bleached (%)	n	Bleached (%)	n
*Diploastrea*	0	3	33	3	0	3
*Psammocora*	0	1	100	2	20	5
*Caulastrea*	0	1	0	1	100	1
*Acropora*	5	21	18	62*	4	83*
*Galaxea*	6	17	20	10	0	14
*Pavona*	21	24	21	14	0	14
*Goniopora*	25	4	50	4	25	4
*Cyphastrea*	40	5	33	3	0	2
*Merulina*	46	54	35	43	8	25
*Astreopora*	50	2	0	3	0	1
*Porites*	53	15	53	17	43	23
*Platygyra*	58	38	61	18	25	24
*Echinopora*	59	22	71	14	36	11
*Favites*	60	20	62	26	20	25
*Euphyllia*	60	5	60	5	100	1
*Goniastrea*	66	41	54	54	11	35
*Montipora*	75	146	64	105	8	64
*Symphyllia*	75	12	83	12	33	12
*Dipsastraea*†	78	9	75	12	22	9
*Podabacia*	80	5	86	7	44	9
*Pocillopora*	83	12	100	10	15	40
*Ctenactis*	83	6	60	5	29	7
*Fungia*	84	25	81	27	20	25
*Hydnophora*	88	8	90	10	25	8
*Pachyseris*	89	35	93	27	44	9
*Pectinia*	89	54	76	41	33	27
*Oxypora*	100	2	100	1	0	2
*Turbinaria*	100	1	50	2	0	3
*Plerogyra*	100	6	100	2	100	3
*Lobophyllia*	100	3	100	2	100	2

^*^Additional surveys of *Acropora* in July and October were carried out by random swims to increase number of colonies surveyed.

^†^This genus was formerly *Favia*.

**Table 2:** Proportion of colonies of different genera exhibiting partial colony mortality.

Partial mortality category							
Genus	n	None	1–10%	11–25%	26–50%	51–75%	76–100%
*Oulophyllia*	1	0	100	0	0	0	0
*Goniastrea*	20	20	20	60	0	0	0
*Lobophyllia*	3	33	0	0	67	0	0
*Merulina*	67	40	4	39	16	0	0
*Diploastrea*	13	54	31	0	0	15	0
*Montipora*	80	64	5	13	16	3	0
*Platygyra*	17	65	18	18	0	0	0
*Echinopora*	17	65	29	6	0	0	0
*Favites*	3	67	33	0	0	0	0
Dipsastraea*	4	75	0	0	0	25	0
*Porites*	22	77	5	18	0	0	0
*Pavona*	26	81	4	15	0	0	0
*Pectinia*	24	83	8	0	4	4	0
*Podabacia*	9	89	11	0	0	0	0
*Pachyseris*	28	96	0	0	4	0	0

^*^This genus was formerly *Favia*.
