# Supplementary material for: Coral community response to bleaching on a highly disturbed reef
Source: Sci Rep. 2016 Feb 15;6:20717. doi: 10.1038/srep20717 (PMC4753424; doi:10.1038/srep20717)
Supplement: Supplementary Information [file srep20717-s1.pdf]

Coral community response to bleaching on a highly disturbed reef

JR Guest*, J Low, K Tun, B Wilson, CM Ng, D Raingeard, KE Ulstrup, JTI Tanzil, PA Todd, TC Toh, D McDougald, LM Chou, PD Steinberg

Supplementary material

Table S1. Proportion (%) of colonies not bleached, moderately bleached, severely bleached and bleaching indices for all genera that had at least 5 colonies surveyed on all three survey occasions.

	June					July					October				
**Genus**	**n**	**Not bleached**	**Moderate**	**Severe**	**BI**	**n**	**Not bleached**	**Moderate**	**Severe**	**BI**	**n**	**Not bleached**	**Moderate**	**Severe%**	**BI**
Acropora	*21*	95	5	0	**2**	*62*	82	18	0	**9**	*83*	96	4	0	**2**
Galaxea	*17*	94	6	0	**3**	*10*	80	20	0	**10**	*14*	100	0	0	**0**
Pavona	*24*	79	17	4	**13**	*14*	79	21	0	**11**	*14*	100	0	0	**0**
Merulina	*54*	54	43	4	**25**	*43*	65	35	0	**17**	*25*	92	8	0	**4**
Favites	*20*	40	50	10	**35**	*26*	38	50	12	**37**	*25*	80	16	4	**12**
Echinopora	*22*	41	41	18	**39**	*14*	29	64	7	**39**	*11*	64	36	0	**18**
Favia	*9*	22	78	0	**39**	*12*	25	58	17	**46**	*9*	78	22	0	**11**
Platygyra	*38*	42	37	21	**39**	*18*	39	28	33	**47**	*24*	75	21	4	**15**
Goniastrea	*41*	34	44	22	**44**	*54*	46	39	15	**34**	*35*	89	6	6	**9**
Porites	*15*	47	13	40	**47**	*17*	47	18	35	**44**	*23*	57	17	26	**35**
Symphyllia	*12*	25	42	33	**54**	*12*	17	75	8	**46**	*12*	67	25	8	**21**
Pocillopora	*12*	17	58	25	**54**	*10*	0	80	20	**60**	*40*	85	15	0	**8**
Ctenactis	*6*	17	50	33	**58**	*5*	40	0	60	**60**	*7*	71	29	0	**14**
Montipora	*146*	25	29	46	**60**	*105*	36	15	49	**56**	*64*	92	6	2	**5**
Hydnophora	*8*	13	50	38	**63**	*10*	10	60	30	**60**	*8*	75	25	0	**13**
Pectinia	*54*	11	52	37	**63**	*41*	24	54	22	**49**	*27*	67	26	7	**20**
Fungia	*25*	16	40	44	**64**	*27*	19	52	30	**56**	*25*	80	16	4	**12**
Podabacia	*5*	20	20	60	**70**	*7*	14	14	71	**79**	*9*	56	44	0	**22**
Pachyseris	*35*	11	17	71	**80**	*27*	7	7	85	**89**	*9*	56	33	11	**28**

Table S2. Change in relative cover (%) of coral genera at Pulau Satumu between 2009 (before bleaching), 2010 (during bleaching) and 2012 (after bleaching). Genera are in order from the greatest reduction in relative cover to the greatest increase in relative cover between 2009 and 2012.

**Genus**	**Relative cover (%)**		
	**2009**	**2010**	**2012**
Porites	8.9	10.8	2.5
Pavona	9.0	9.5	3.8
Pachyseris	7.4	3.2	2.6
Montipora	13.8	10.3	9.8
Echinopora	4.4	3.6	1.0
Pectinia	6.7	6.6	4.1
Hydnophora	3.0	1.4	0.7
Symphyllia	2.3	2.0	0.9
Goniopora	1.0	0.6	0.0
Leptoseris	0.5	0.3	0.0
Lithophyllon	0.5	0.0	0.0
Fungia	0.9	1.4	0.5
Herpolitha	0.5	0.0	0.3
Lobophyllia	0.1	0.4	0.0
Heliopora	0.2	0.0	0.2
Coscinaraea	0.0	0.7	0.0
Euphyllia	0.0	1.0	0.0
Turbinaria	0.0	0.5	0.0
Ctenactis	0.5	0.4	0.6
Acropora	0.7	2.4	0.8
Cyphastrea	0.6	0.1	0.8
Mycedium	0.2	0.0	0.5
Leptoria	0.0	0.2	0.4
Favites	3.4	4.3	4.3
Psammocora	0.3	0.5	1.4
Merulina	16.5	19.3	17.7
Acanthastrea	0.0	0.0	1.5
Pocillopora	0.2	0.1	1.8
Galaxea	0.7	1.0	2.5
Favia	0.6	2.0	2.4
Oulophyllia	1.0	0.3	2.9
Goniastrea	6.4	7.6	8.6
Podabacia	0.3	1.6	2.7
Echinophyllia	1.5	0.2	4.2
Platygyra	5.1	5.9	8.3
Diploastrea	2.8	1.9	12.5

Symbiont clade analysis: Methods and materials

Coral samples were immediately snap-frozen at -80°C after collection and tissue samples were directly resuspended in the Tris-EDTA buffer. DNA was extracted from all samples using the phenol-chloroform method[1]. DNA yield and purity were assessed using a NanoDrop 1000 spectrophotometer (Thermo Scientific, USA) and sent to the Research and Testing Laboratory (RTL, USA) for PCR amplification (using Primers ITSDF (5'-GTGAATTGCAGAACTCCGTG-3') and ITS2-R (5'-CCTCCGCTTACTTATATGCTT -3')[2] for the ITS2 region between the 5.8S and 28S rRNA subunits of *Symbiodinium*) and pyrosequencing using the Roche 454 FLX Titanum platform (Roche Diagnostics Corporation, USA). A total of 3556 reads with a median length of 320 bp were obtained from sequencing and processed using the QIIME pipeline (Version 1.8.0)[3]. Briefly, reads were trimmed (> 150 bp and *phred* quality score ≥ 20)[4] and demultiplexed using the split_libraries.py script. The pick_otus.py script was used for *de novo* OTU picking, using cd-hit[5] and a sequence similarity threshold of 97%, and representative OTUs selected using the pick_rep_set.py script. Representative OTUs were aligned with the GeoSymbio database[6] using NCBI BLAST+ tools[7] and the putative cladal or subcladal ID and percentage identity of the top hit for each OTU sequence parsed. Pyrosequencing data were submitted to the NCBI Sequence Read Archive (SRA) under Accession Number SRP068395, whilst sequence data for the representative OTUs listed in Table S3 were submitted to NCBI GenBank under Accession Numbers KU549088-KU549107.

Table S3. Relative proportions of *Symbiodinium* OTUs and putative cladal or subcladal identification for independent tissue samples of *Platygyra sinensis* and *Pocillopora damicornis* collected in 2012 from sites south of Singapore.

	Sample ID[a]						
OTU#	Pd1 K<br>*n* = 90	Pd2 K<br>*n* = 2510	Pd3 PS<br>*n* = 60	Ps1 PS<br>*n* = 665	Ps2 PS<br>*n* = 182	Ps3 PS<br>*n* = 49	Putative ID[b]<br>(% Identity)
4						53	C3 (99)
6	13	17	92	33		12	D1 (99)
7	41	5	3	9	17		D1 (99)
8	7	2		11	4	2	D1 (98)
9					2		D1 (99)
11	3	1		4	19	2	D1 (99)
12		12					D (100)
15						2	C3 (98)
16	19	5			33		D1 (99)
17	1			11		12	D (99)
18		7		13			D (99)
20		2			3	4	D1 (100)
21	1	15					D (99)
23	12	4	5	10	15	12	D1 (99)
24				6			D (100)
25				1			D (99)

26	2	24					D (99)
27		1		2	2		D1 (99)
28		5					D (99)
31					3		D1 (99)

[a] Sample ID = {Species}{Replicate}{Site}, where:

{Species} Pd=*Pocillopora damicornis*; Ps= *Platygyra sinensis;*
{Replicate} 1,2 or 3;
{Site} K = Kusu, PS = Pulau Satumu;
n = Number of sequence reads per sample after quality trimming.

[b]Putative subcladal ID assigned from top BLAST+ hit to GeoSymbio[6] database. In the event that taxonomy could not be assigned to subcladal level, cladal affiliation was used.

References

1 Sambrook, J., Russell, D.W. Purification of Nucleic Acids by Extraction with Phenol:Chloroform. *Cold Spring Harbor Protocols*. doi: 10.1101/pdb.prot4455 (2006).

2 Pochon, X., Pawlowski, J., Zaninetti, L., Rowan, R. High genetic diversity and relative specificity among *Symbiodinium*-like endosymbiotic dinoflagellates in soritid foraminiferans. *Mar. Biol.* **139,** 1069–1078 (2001).

3 Caporaso, J.G. et al. QIIME allows analysis of high-throughput community sequencing data. *Nature Methods*. **7,** 335-336 (2010).

4 Quigley, K.M., Davies, S.W., Kenkel, C.D., Willis, B.L., Matz, M.V., Bay, L.K. Deep-Sequencing Method for Quantifying Background Abundances of *Symbiodinium* Types: Exploring the Rare *Symbiodinium* Biosphere in Reef-Building Corals. *PLoS ONE*. **9(4),** e94297 (2014).

5 Li, W. & Godzik, A. Cd-hit: a fast program for clustering and comparing large sets of protein or nucleotide sequences. *Bioinformatics*. **22,** 1658-1689 (2006).

6 Franklin, E.C., Stat, M., Pochon, X., Putnam, H.M., Gates, R.D. GeoSymbio: a hybrid, cloud-based web application of global geospatial bioinformatics and ecoinformatics for *Symbiodinium*–host symbioses. *Mol. Ecol. Res.* **12,** 369-373 (2012).

7 Camacho, C., Coulouris, G., Avagyan, V., Ma, N., Papadopoulos, J., Bealer, K., Madden, T.L. BLAST+: architecture and applications. *BMC Bioinformatics* **10**, 421 (2009).
